# Rethinking the Use of Mobile Apps for Dietary Assessment in Medical Research

**DOI:** 10.2196/15619

**Published:** 2020-06-18

**Authors:** Wael Khazen, Jean-François Jeanne, Laëtitia Demaretz, Florent Schäfer, Guy Fagherazzi

**Affiliations:** 1 Kelly OCG Paris France; 2 Innovation Science and Nutrition Danone Nutricia Research Palaiseau France; 3 Digital Epidemiology Hub Department of Population Health Luxembourg Institute of Health Strassen Luxembourg; 4 Center of Research in Epidemiology and Population Health UMR 1018 Inserm, Institut Gustave Roussy Paris-Sud Paris-Saclay University Villejuif France

**Keywords:** diet, dietary assessment, epidemiology, clinical research, mobile diet app, academic apps, consumer-grade apps

## Abstract

Food intake and usual dietary intake are among the key determinants of health to be assessed in medical research and important confounding factors to be accounted for in clinical studies. Although various methods are available for gathering dietary data, those based on innovative technologies are particularly promising. With combined cost-effectiveness and ease of use, it is safe to assume that mobile technologies can now optimize tracking of eating occasions and dietary behaviors. Yet, choosing a dietary assessment tool that meets research objectives and data quality standards remains challenging. In this paper, we describe the purposes of collecting dietary data in medical research and outline the main considerations for using mobile dietary assessment tools based on participant and researcher expectations.

## Diet Assessment in Clinical Studies: Purposes and Limitations

In which situations should diet be assessed in clinical research? Which considerations should drive the selection of a dietary assessment tool (DAT)? This paper addresses these two questions, emphasizing that one should consider not only the research objectives and study setting, but also the opportunities and limitations of available methods for acquiring and analyzing dietary data.

In epidemiological and clinical observational studies, dietary parameters are adequately considered when information on usual dietary intake is needed at a population level [1] or when diet-disease relationships are being explored [2]. In these settings, evaluating dietary intake over time has proved efficient for investigating diet-disease associations at the population level, especially in chronic conditions [2]. Tracking participant lifestyle was needed to understand the role of unhealthy food and diet in the development of overweight, obesity, type 2 diabetes [3], cardiovascular diseases [3,4], and cancer [5]. However, despite the availability of scientifically validated methods to assess dietary intake and usual intake, their use in establishing diet-disease relationships remains controversial [6,7].

In interventional settings, dietary parameters need to be considered for efficacy assessments or to control confounding effects, irrespective of the nature (dietary, drug, or other) of the intervention [8]. However, background diet is, to our knowledge, not systematically assessed in drug development studies despite evidence of the presence of food-drug interactions.

Food-drug interaction is also an important factor to consider during clinical development, as some chemical compounds in foods can potentially affect the pharmacokinetic, pharmacodynamic, or metabolic pathways of some drugs [9]. For example, it is known that leafy green vegetables such as kale and spinach, which contain high levels of vitamin K, can reduce the effectiveness of some oral anticoagulants, such as warfarin [10]. In parallel, we must not neglect how the side effects of medicinal products can influence appetite [11].

## Choosing the Optimal DAT

Selecting a DAT adapted to a research question is usually a matter of compromise. Current methods to assess diet are available in various formats (paper or electronic), to obtain data on dietary intake (prospectively or retrospectively) or usual intake [12]. In a comprehensive resource guide issued by the Food and Agriculture Organization of the United Nations [13] and as explained by other authors [14], various available DATs can be distinguished based on technology (including data acquisition):

- Conventional methods for dietary data collection, which include food records, food frequency questionnaires, 24-hour dietary recalls and diet history, which are widely used in research, with known strengths and limitations
- DATs based on innovative technologies, which are divided into 5 categories according to the acquisition method: personal digital assistant, image-assisted methods (ie, digital cameras), mobile-based technology, interactive computer and web-based technologies, and scan and sensor-based technologies. It must be noted that DATs based on innovative technologies can be digitized versions of the above-mentioned conventional methods.

**Figure 1:**
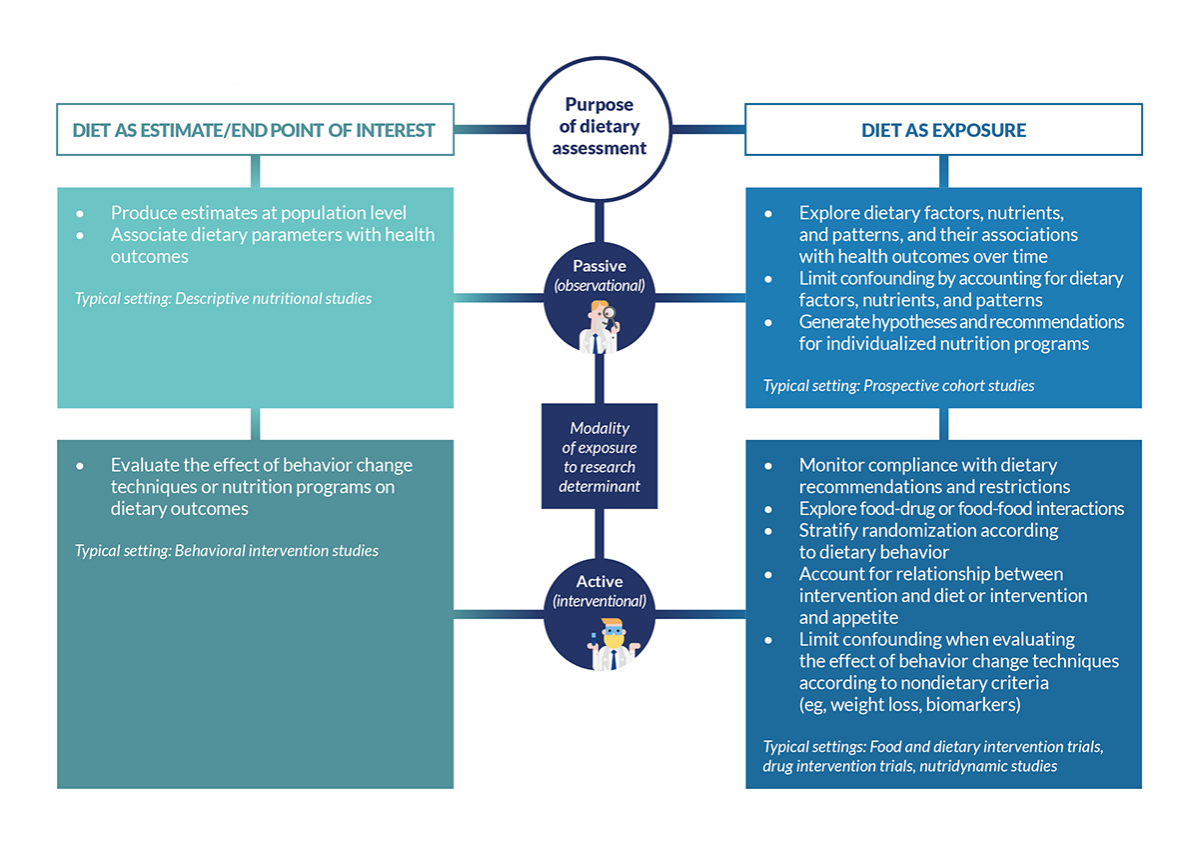
Relevant frameworks for using dietary assessment tools in human studies.

Collecting dietary data is, however, not always needed for primary study analyses, as one may only be interested in monitoring the background diet or compliance with dietary restrictions while focusing on many other clinical and nonclinical parameters (Figure 1).

Because collecting dietary information usually requires substantial involvement from clinical study participants, selecting an appropriate DAT represents a trade-off between participant burden and the volume or accuracy of the collected information. Compromises may include collecting dietary intake data at specific time points rather than tracking each eating occasion, and choosing a reasonable frequency for assessing food intake throughout the research studies.

**Figure 2:**
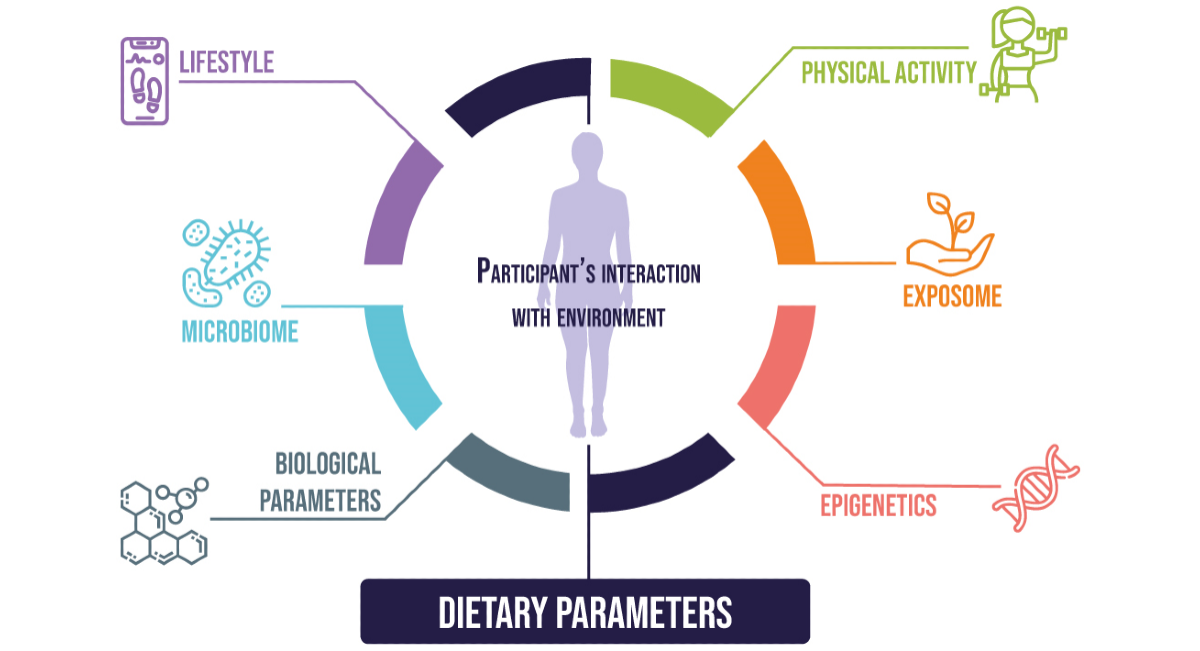
Parameters that can be measured or accounted for along with dietary parameters in medical research.

However, limiting the dietary assessment to habitual food intake (evaluated using food frequency questionnaires) is not relevant for every study, especially when associations between dietary and biological parameters are investigated, as outlined in Figure 2.

Instead of evaluating habitual food intake, daily tracking of each eating occasion may be needed. One example is the daily variation of gut microbiota composition, known to be related to food choice [15], requiring, when investigated, exhaustive and daily tracking of a participant’s dietary intake. Handling temporality, therefore, represents a major challenge when selecting a relevant DAT, in case there is important intrasubject variability of parameters over time. We believe that a focus is needed for identifying or developing solutions that can effectively track participants’ background diet throughout their participation in research studies (observational or interventional), irrespective of the intervention (drug, food, or dietary) while limiting the associated participant burden. DATs based on new technologies offer opportunities to facilitate traditional food intake measures, and mobile apps have demonstrated being adequate for both participants and researchers. For participants, mobile apps represent an acquisition method that is acceptable, accessible, ubiquitous, and one that can be used by participants alone [16-18]. For the study sponsors or researchers, these apps offer other opportunities in terms of cost-effectiveness, reducing time between the collection and reporting of dietary data, and improving data quality with an accurate, comprehensive, and relevant food composition data set [14].

Mobile apps, therefore, represent a good compromise for researchers (investigators and sponsors) when the collection of dietary data is needed.

## The Need to Explore the True Potential of Mobile Apps as DATs

When navigating the astronomical number of mobile diet apps (approximately 30,000 diet and physical activity mobile apps are currently available on Google Play and the App Store), selecting a DAT that fits with research purposes remains a key challenge. This selection should be based on multiple parameters, including the suitability of user experience and interface, the reliability of the food composition database (FCD), the relevance of the data acquisition method (identification and quantification of foods), and the quality of nutritional outputs.

To compare these mobile diet apps in terms of reliability, quality, and effectiveness, we cannot consider the number of downloads as a reliable evaluation parameter, as the most frequently downloaded apps are not necessarily the most usable or efficient in medical research [19]. Neither can we assess these tools based on user ratings (ie, stars), which are known to be biased [20]. Currently, no official standards exist for evaluating health-related mobile apps, making the evaluation of the strengths and weaknesses of these apps complex for researchers when choosing a relevant tool [21].

High-quality reviews and meta-analyses are few, and there is limited comparability between the evaluation methods used. Some of the reviews evaluate functionality, information quality, esthetics, and engagement [22]; others evaluate the accountability, usability, scientific coverage, and technology-enhanced features based on the opinions of nutrition experts who had tested different apps for 5 days [23]. In parallel, other experts have developed their own scales for a qualitative assessment of mobile health apps, including the following:

- Mobile App Rating Scale, which focuses on 4 evaluation parameters by an expert panel, including engagement, functionality, aesthetics, and quality of the information provided by the app [24]
- App Quality Evaluation, which evaluates the educational quality and technical functionality of nutrition apps by nutrition professionals and app users, based on several parameters, including app function, purpose, behavior change potential, support of knowledge acquisition, and skill development [25]

To our knowledge, only 3 studies have compared the nutritional values provided by different mobile diet apps based on quantitative parameters. One research team evaluated the accuracy of 7 diet apps in providing nutrient values. In their study, the nutrient labels of 100 food products available in the Netherlands were compared to the nutrient values provided in the nutrition facts in each app. The researchers concluded that the accuracy of nutrient values varied enormously between these apps, and energy was the most reliable value [26]. Another research team compared the nutrient intake calculations of 5 consumer-grade mobile diet apps to the Nutrition Data System for Research dietary analysis program, concluding that most of the nutritional values (except energy and some macronutrients) provided in these apps are generally underestimated, and that some apps are more accurate than others [27].

More recently, another team evaluated energy, macronutrient, and available micronutrient values provided by 5 popular nutrition apps against a UK reference method (Dietplan6 [28]). The authors concluded that the values of energy, carbohydrates, saturated fats, total fat, and fiber reported by these apps (except for 1 app) were rather reliable, while the values of protein, sodium, and micronutrients were inconsistent and less reliable [29].

## Academic and Consumer-Grade Mobile Diet Apps

### Two Main Categories of Mobile Diet Apps

Even though mobile diet apps are emerging tools, 2 main categories can be distinguished based on the affiliation of the developers and their objectives:

- Academic apps, developed by experts in nutrition or dietetics to provide a reliable, scientifically validated tool; these are mainly developed for research purposes. Known examples include Technology Assisted Dietary Assessment [30], DietCam [31], and My Meal Mate [32].
- Consumer-grade apps that are service-oriented, typically developed by private entities specialized in digital development [33]. These apps are intended to be used by the lay public, and their purpose is mainly commercial. Popular examples include MyFitnessPal, FatSecret, and Lifesum. Weight management is usually the main feature of this second category of apps.

**Figure 3:**
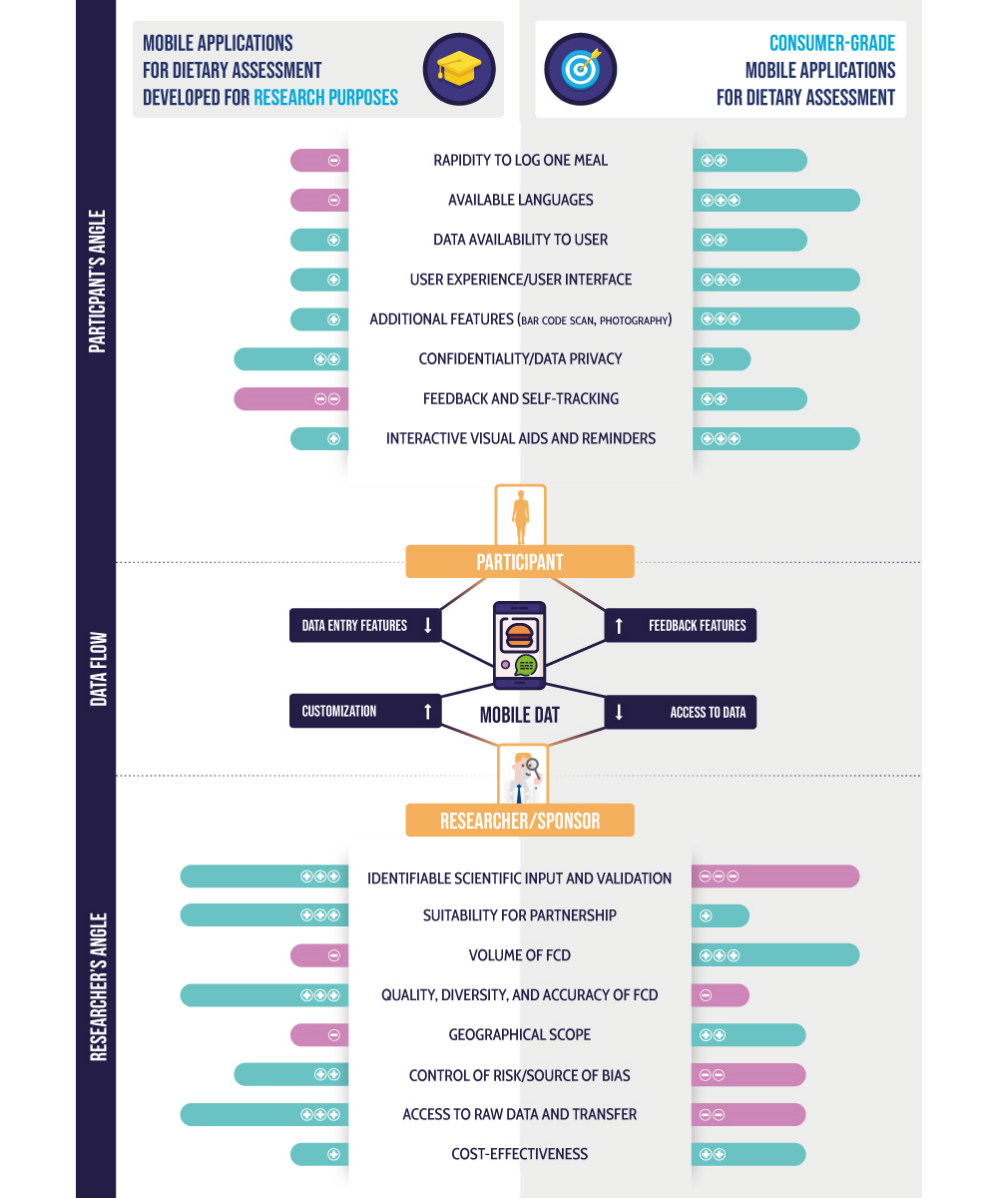
Key aspects of mobile dietary assessment tools (DATs) developed for research purposes and consumer use from the participant and researcher perspectives. FCD: food composition database.

The main strengths and limitations of each category, from the participant and researcher perspective, are outlined in Figure 3.

### Academic Apps: A Focus on Scientific Validation

From a researcher's point of view, academic apps offer more advantages than consumer-grade apps, as they are usually developed with identifiable scientific input and compared with a standard method for validation purposes. Typical examples of academic apps include Electronic Dietary Intake Assessment (e-DIA) [34] and My Meal Mate [32]. Academic apps are primarily focused on research and do not aim to be popular. For most of these apps, the number of users is unknown (as they are usually not referenced in Google Play or the App Store), but we assume it is lower than the number of users of consumer-grade apps, which aim to be visible in the stores.

Another main advantage of academic apps is the absence of any constraints in terms of advertising and commercial incentives for users. We also believe that these apps are safer in terms of privacy and confidentiality than those designed for consumer use.

A recent systematic assessment of technology-based DATs [35] compared the features of research and consumer tools. The authors concluded that features facilitating data entry (including voice, digital images, and bar code scan) were more frequently available in “consumer” apps, such as DietCam or My Meal Mate. This work, however, did not consider the “consumer-grade” apps that are discussed in this paper. Other examples of academic apps or apps developed with identifiable academic input that include such features are MyFoodRepo (which includes an image recognition algorithm) [36] and FODMAP App for people with irritable bowel syndrome (FoodMaestro bar code scan, a commercial app) [37]. Most academic apps are not designed for universal use as they are validated for specific homogenous populations, which prevents their use in an international clinical study. Similarly, another important difference between these 2 categories is the number of available languages, as most academic apps are usually available in 1 or 2 languages at most (myfood24 [38] is available in English, German, and Danish, and developers are currently working on the release of an Arabic version).

The question of the scientific validation of academic apps remains a matter of debate. In general, academic apps are evaluated using another self-report instrument thought to capture diet as a reference, such as 24-hour dietary recalls. The choice of the reference method can be controversial, as stated by the National Cancer Institute, which considers that data collected using this method contain errors, including intake-related bias [39]. It should be noted that the e-DIA app was validated by comparison to 24-hour dietary recall as a reference [34], and the same reference method was used to validate My Meal Mate app [32]. Results of validation studies must, therefore, be interpreted with caution when scientific validation is relative and not absolute. These validation methods are less reliable than using gold-standard reference measures, such as biomarker recovery, or methods to capture true intake without systematic errors, such as direct observation and feeding studies [39].

### Consumer-Grade Apps: Volume of FCD Data and User Experience

Given the lucrative purpose of consumer-grade apps, an app must be attractive to appeal to a maximum number of users. Even though this environment is competitive, some of these apps are very popular and are downloaded tens of millions of times around the world (such as MyFitnessPal, FatSecret, and Lose It!). For the end user, the apps designed as a service usually offer an appealing user interface and experience: their design is attractive, which facilitates the identification of a meal and may reduce the time required to enter data compared to some academic apps. Another major difference between these 2 categories is the number of languages available (for example, MyFitnessPal is available in 19 languages [26]). The use of a very rich and international FCD is usually a key differentiator among these apps. However, the quality of the FCDs, regularly pointed at as a major issue with consumer-grade apps, is discussed later in this article.

The usability of consumer-grade apps is now being explored but is difficult to assess. A recent review [40] concluded that the features available in consumer apps vary greatly, and there are additional variations between Android and iOS versions. The key features of these apps usually include interactive visual aids and reminders, which are available in basic free versions (even though some apps require subscriptions) to help users achieve their goals. Thus, to further engage users in improving these apps, end-users can add new food products that are not available in the FCD, including energy and nutrient values, or to correct inaccurate information [41]. This may be beneficial for FCD enrichment, but it may also be a major and dangerous source of error in data entry. The addition of new food products and associated nutrient values by lay users can lead to input errors, especially when the developers of these apps do not control the quality and integrity of the data sets, and this is one of the most important negative features of consumer-grade apps.

On the other hand, the use of these consumer-grade apps in medical research has certain limitations and risks, especially with regard to data privacy and confidentiality. The lack of documentation regarding the privacy of user data may represent a major challenge for the legal and ethical integrity of personal and food data [42]. A recent example is the data breach of 150 million accounts of MyFitnessPal [43], which is considered the world's no. 1 app in the field of consumer-grade diet-tracking apps. Another major limitation of these apps is the strong incentive to purchase a paid version through advertisements and recurring notifications: these may cause annoyance to the users of a basic free version and represent a bias in research when too many recommendations are provided as they may interfere with evaluation of the parameters.

We can safely assume that most consumer-grade apps have been developed without rigorous scientific validation and do not systematically involve nutrition experts during development. A recent review of 28 905 relevant health-oriented mobile apps showed that only 17 apps (0.05%) were developed with identifiable professional input [44]; other analyses showed that only 0.8% (3/393) of weight management apps have been scientifically evaluated [45].

Consumer-grade apps may, however, offer some benefits to researchers, such as FCD data volume, as it differs hugely between these 2 categories of apps. In academic apps, the approximate number of food items in the FCD is tens of thousands at most (8500 for DietCam [31] and 40,000 for My Meal Mate [35]), while this number is much higher in several consumer-grade apps (eg, 18 million foods claimed by MyFitnessPal developers [46]).

### Room for Improvement of Consumer-Grade Apps

If the volume of FCD data is a key differentiator for these 2 categories of apps, the quality, diversity, and accuracy of FCDs is widely in favor of academic apps. Indeed, the quality of FCDs of consumer-grade apps remains questionable, as explained by several authors [26,27,29] who have concluded that these apps lack a reliable FCD; provide a limited set of nutrients to users; are too focused on energy and macronutrient intake, with a lot of missing data; and underestimate macronutrient and micronutrient values.

Several developers assume that some food information in their database is inaccurate, and some of them, such as MyFitnessPal [41] or Lose It! [47], invite users to correct these errors using crowdsourcing techniques.

Academic apps are regarded as more reliable in their FCD, based on objective measurements and scientific studies [48,49]. For researchers and sponsors, other benefits are in favor of academic apps, including the suitability for partnership, as the developers of these types of apps are usually more open to share information about the development and validation of these tools. Considering that these apps are primarily designed for research, the access to and transfer of raw data are facilitated for researchers with dedicated access. For apps focused on consumers, the access to data is mainly provided to users, especially those who subscribe to optional features. In addition, the absence of feedback features in academic apps reduces the risk of bias in the tracking of eating occasions. However, it should be noted that the geographic scope of consumer-grade apps, which are developed and available worldwide, is far more significant than that of academic apps, which are usually limited to one or two countries.

## Discussion

In summary, we can surmise that researchers will usually favor using academic mobile apps, while end users may be more attracted to the features offered by consumer-grade mobile apps. Based on our experience and the cited opinions of experts in the field of nutrition, we understand that currently, there is no one-size-fits-all solution for tracking dietary intake that can be used in every type of medical research, as each available tool has its limitations [35,50] due to language availability, validation in specific populations, and the reliability of FCDs. The ideal option would be to develop a mobile app for tracking the diet that has the strengths of both types of apps: an efficient, user-friendly interface and experience, coupled with an FCD that is as rich as it is reliable. In the absence of such a tool, or in developing one soon, we must select the most appropriate one for each type of study according to its objectives. The landmark DIET@NET (DIETary Assessment Tool NETwork) suggests considering 5 steps before choosing the appropriate type of DAT, and the first step is, of course, to define the research question well [50].

If the daily use of consumer apps by millions of people worldwide can generate big data on food consumption, this information can be used by researchers (while complying with data privacy requirements) to study health, diet, and dietary behavior parameters. This can be done by considering cultural aspects of populations at different levels (country, household, or individual) while integrating potential key cofactors such as physical activity level, sleep, weight, or location data, which can often be collected simultaneously. It has also been highlighted that the most frequently downloaded consumer-grade apps lack features relating to emotions, even though emotions are known to be associated with diet [40]. Using these diet apps as a reliable food-tracking tool in clinical trials or research, in general, is a major challenge, considering their limitations; despite the popularity of these apps, the lack of evidence and scientific validation of their use remains the major issue [51]. Little information is available on the quality of these mobile apps, other than what can be gathered from users’ ratings published on mobile app stores. The lack of reliability of the data these apps provide, especially in the FCD, is a real barrier for researchers who want to utilize the user data. It should also be noted that the legal and ethical concerns related to user data for consumer-grade mobile health apps are well documented in the literature [52-54]. A recent review showed that most mobile diet apps do not provide terms of use or privacy policy documents [42].

The use of consumer diet apps in studies should be done with caution: given the nature and suboptimal quality of their FCDs, their use should be limited to the tracking of some macroelements such as energy intake. Energy intake is considered the most consistently and accurately value reported by these apps compared to other macro- and micronutrients [23,26], which are generally underestimated [27,29,55]. Recent studies have also shown that different consumer apps, such as Samsung Health, MyFitnessPal, and FatSecret, provide an acceptable estimate of energy, carbohydrate, and fat intake [29], and that MyFitnessPal offers a good relative validity for energy and fiber tracking [55]. Many dieticians (in the United Kingdom, New Zealand, and Australia) use nutrition apps in their practice [56]. However, for many experts in the field of nutrition, mobile apps designed for consumer awareness are an unreliable source of nutritional values due to the crowdsourcing nature of the FCD. This issue is leading to a lack of interest in using these new tools for nutritional and clinical studies.

## Perspectives

Our opinion is that the potential of mobile apps designed for consumer use has not been sufficiently evaluated, especially in clinical settings. Despite the limitations that have been outlined above, we believe that the data collected by these apps represent a source of information we cannot ignore, as it may be sufficient in some epidemiological or clinical studies, when the assessment of diet is not the primary objective of a research study. Potential uses of these tools include segmenting participant populations into subgroups according to usual dietary intake or associating specific meal patterns with biological parameters during a short time frame. However, in our view, further work is needed to assess this potential. We should also consider the potential of other features that are now included in several mobile apps, such as automated, image-based recognition. This feature, while promising, is only in an early stage of development, and there is still room for improvement until it can be used regularly by clinical study participants. However, when relevant, analytical methods offered by artificial intelligence may soon be used to obtain both qualitative (ie, composition of meals) and quantitative (ie, portion sizes) outputs. We believe that such features may one day be the bridge that accommodates participants and researchers, filling the gap between academic and consumer-grade apps once they are available in both types of solutions.

## Abbreviations

DAT: dietary assessment tool
FCD: food composition database
